# Specialized Diagnostic Investigations to Assess Ocular Status in Hypertensive Diseases of Pregnancy

**DOI:** 10.3390/diseases4020019

**Published:** 2016-04-22

**Authors:** Rahul Navinchandra Bakhda

**Affiliations:** Consultant Ophthalmologist, Ex-Resident, M & J Western Regional Institute of Ophthalmology, Ahmedabad 380016, Gujarat, India; dr_rnbakhda@zoho.com; Tel.: +91-281-2475297

**Keywords:** hypertensive diseases of pregnancy, preeclampsia, eclampsia, investigations

## Abstract

This review describes specialized diagnostic investigations to assess ocular status in hypertensive diseases of pregnancy. Ocular assessment can aid in early detection for prompt multidisciplinary treatment, obstetric intervention and follow-up. The investigations accurately predict the possible causes of blindness in hypertensive diseases of pregnancy. The investigations include fluorescein angiography, ophthalmodynamometry, fluorophotometry, imaging modalities, OCT, ultrasonography, doppler velocimetry and blood chemistry analysis. The review includes a summary of imaging techniques and related recent developments to assess the neuro-ophthalmic aspects of the disease. The imaging modalities have been instrumental in understanding the complex neuropathophysiological mechanisms of eclamptic seizures. The importance of blood chemistry analysis in hypertensive diseases of pregnancy has been emphasized. The investigations have made a significant contribution in improving the standards of antenatal care and reducing maternal and fetal morbidity and mortality.

## 1. Introduction

Hypertensive diseases in pregnancy are important causes of maternal and fetal mortality across the globe [1,2,3]. Hypertensive diseases of pregnancy are characterized by functional derangements of multiple organs. The eye and visual system are no exception. Ocular symptoms are frequent [4]. Visual symptoms may be the precursors of seizures. Ocular assessment can aid in early detection for prompt multidisciplinary treatment, obstetric intervention and follow-up. This review describes the specialized investigations to assess ocular status in specific situations apart from clinical examination of the eye, *i.e.*, torch light examination, ophthalmoscopy and essential laboratory investigations [5].

The specialized investigatve modalities in hypertensive diseases of pregnancy are as follows:

## 2. Fluorescein Angiography and Indocyanine Green Angiography in Hypertensive Diseases of Pregnancy

### 2.1. Fluorescein Angiography

**Figure 1:**
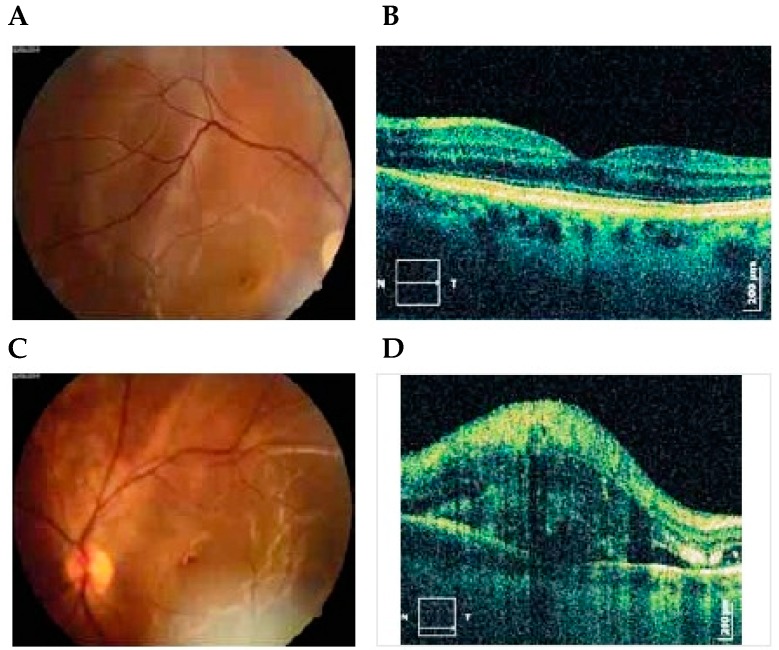
Photographs of right (**A**) and left (**C**) eyes, 1 day after delivery showing bullous serous retinal detachment. OCT exams of right (**B**) and left (**D**) eyes 5 days after delivery demonstrated subretinal and intraretinal fluid more expressed in the left eye [7]. Published with permissions of the publisher-The Association of Basic Medical Sciences of Federation of Bosnia and Herzegovina.Original source: Sreckovic, S.B.; Janicijevic-Petrovic, M.A.; Stefanovic, I.B.; Petrovic, N.T.; Šarenac, T.S.; Paunovic, S.S. Bilateral retinal detachment in a case of preeclampsia. *Bosn J Basic Med Sci* **2011**, *11*, 129. © Association of Basic Medical Sciences of FBIH, 1998-2016. Credits to Public Knowledge Project.

**Figure 2:**
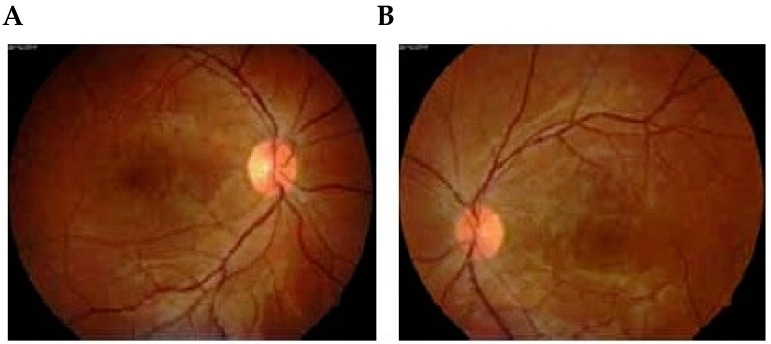

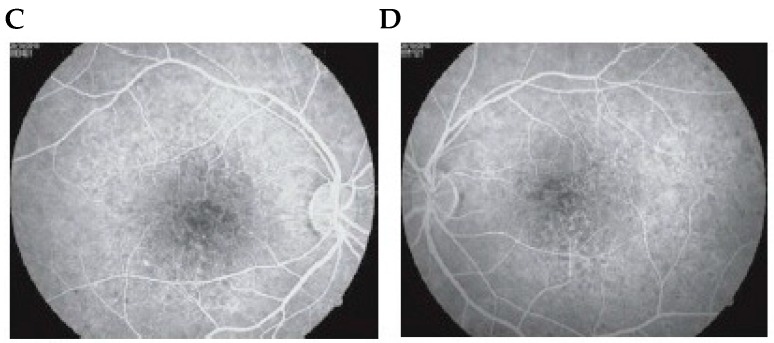
Follow-up examination 1 month later showed total resolution of bilateral serous retinal detachment of right (**A**) and left (**B**) eyes. (**A**) Fluorescein angiogram performed on the same day showed focal pigmentary abnormalities in areas where there had been a detached retina of right (**C**) and left (**D**) eyes [7]. Published with permissions of the publisher-The Association of Basic Medical Sciences of Federation of Bosnia and Herzegovina.Original source: Sreckovic, S.B.; Janicijevic-Petrovic, M.A.; Stefanovic, I.B.; Petrovic, N.T.; Šarenac, T.S.; Paunovic, S.S. Bilateral retinal detachment in a case of preeclampsia. *Bosn J Basic Med Sci* **2011**, *11*, 129. © Association of Basic Medical Sciences of FBIH, 1998-2016. Credits to Public Knowledge Project.

Fluorescein angiographic studies, although limited, have been helpful in interpreting the abnormal retinal and choroidal vascular changes that occur in preeclampsia [6]. Fluorescein angiography in patients without retinal detachment exhibit retinal vascular decompensation manifested by dilated capillaries, widespread capillary occlusion and perivascular and optic nerve head staining. The majority of fluorescein angiographic studies have emphasized the absence of retinal vascular abnormalities and demonstrated choroidal vascular abnormalities in preeclamptic patients with retinal detachment. Figure 1 is a fundus photograph of a preeclamptic patient with bullous serous retinal detachment [7,8]. Delayed perfusion of choriocapillaries in the early phase of angiogram was observed, and, in middle and late phases, areas of non-perfusion showed foci of gradual fluorescein leakage with coalesence of dye in the subpigment and subretinal space. These observations provided clinical evidence that retinal detachment in preeclampsia was secondary to choroidal arteriole and chorio capillaries occlusion. Following resolution of exudative detachment, irregular alteration of pigment epithelium was noted in the macula and posterior pole, as illustrated by fluorescein angiograms in Figure 2 [7]. The focal areas of depigmentation or hyper pigmentation were the result of healed ischemic infarcts of choriocapillaries (Elschnig’s spots) and accounted for the transmission or blockage of fluorescein dye [9]. Fluorescein angiographic studies of toxemic patients with serous retinal detachment have been limited because of teratogenic effects on the fetus. OCT angiography is an emerging non-invasive imaging modality for visualization of retinal micro vasculature by repeated scans in the same location at different points of time using a split-spectrum amplitude decorrelation angiography (SSADA) algorithm [10].

### 2.2. Indocyanine Green Angiography

Indocyanine green angiography (ICG) offers the advantage of enhanced imaging of the choroidal circulation, as compared to fluorescein angiography [11]. Recent developments, *i.e.*, videoangiography and scanning laser ophthalmoscopy, have increased the utility of ICG in clinical studies [12]. ICG is a tricarboncyanine dye which is highly protein-bound and does not escape from the choriocapillaries. ICG absorbs and reflects light in the near infrared portion of the spectrum, *i.e.*, 805 nm and 835 nm, respectively. It facilitates visualization of the choroid through hemorrhage or other pigmentary deposits of the retina of RPE.

There is a lot of hesitation and reluctance among health care professionals regarding the use of fluorescein and ICG angiography in pregnant patients in spite of the documented safety, particularly of ICG angiography and intravenous ICG to measure hepatic blood flow in pregnant patients [13,14]. According to Fineman *et al.*, “both ICG and fluorescein are classified by the Food and Drug Administration as pregnancy category C, indicating that studies have not been conducted and therefore it is unknown whether fetal harm will result when administered to a pregnant woman [14].” In a majority of centers, fluorescein angiography and indocyanine green angiography have been done after delivery of the fetus and termination of pregnancy to avoid any possible adverse effects to the fetus or the mother.

Confocal laser scanning angiography allows for simultaneous fluorescein and ICG angiography [15]. Compared with consecutive investigations using both dyes, simultaneous angiography requires only one injection, is not associated with additional side effects, and offers identical digital frames.

## 3. Ocular Fluorophotometric Findings in Toxemia of Pregnancy

Ocular fluorophotometry (OFP) is a useful clinical tool for evaluating early disruption of the blood ocular barrier in vascular disease such as toxemia of pregnancy [16]. Ocular fluorophotometry is a more sensitive technique for demonstrating early leakage from retinal vessels than fluorescein angiography [17]. In toxemia of pregnancy, a blood-aqueous barrier deteriorates earlier than blood-retinal barrier. Discrete arterial narrowing is difficult to recognize on fundus examination and might be easily seen on red-free photographs or early phase fluorescein angiograms. Ocular fluorophotometry has been instrumental in recognizing early abnormalities of arterial diameter and quantifying blood ocular barrier disruption. Fluorescein concentrations in the aqueous and posterior vitreous increased significantly in toxemic patients compared with those in normal subjects [18]. There have been promising results currently on the use of fluorophotometry as a non-invasive route of drug delivery via subconjuctival and intravitreal routes [19].

## 4. Ophthalmodynamometry in Toxemia of Pregnancy

Bailliart has made significant contribution in the field of ophthalmodynamometry [20]. He was also the first to describe the relationship between retinal and brachial arterial pressure, the normal ratio being 0.45:1 for the diastolic and 0.54:1 for systolic pressure. He pointed out that in hypertensive conditions, the retinal diastolic pressure rise in proportion to rise in brachial diastolic pressure. The rise in retinal systolic pressure however, is proportionately greater than the rise in brachial systolic pressure, the proportion being 0.7 to 0.8:1 [21,22]. The arteriolar pressure in the central nervous system is well reflected in arteriolar pressure of retina. It was with the possibility of detecting very early signs of hypertension in pregnancy; studies regarding ophthalmodynamometry measurement of retinal arterial pressure were undertaken. In toxemia of pregnancy a comparatively greater rise of diastolic retinal pressure should be looked upon with special concern; as it is in these cases that eclampsia may develop. An increase in diastolic retinal pressure, calls for a careful and regular prenatal survey; and perhaps termination of pregnancy to prevent vascular damage; with detriment to maternal health. In the absence of eye ground changes; however the pregnancy may be continued if kept under constant supervision. The systolic pressure in the retinal artery shows an erratic trend and therefore we do not attach much importance to this change. Retinal diastolic pressure is betters indication of probable organic vascular changes. It can thus be concluded that there is a disproportionate rise in the retinal diastolic pressure in preeclampsia and in eclampsia; the greater the disproportion, the graver the prognosis. Ophthalmodynamometry is recommended a routine technique for investigation in pregnancy. New modification in ophthalmodynamometry device consists of pressure sensor with mounting support of Goldman contact lens [23]. Ophthalmodynamometry has shown a lot of promise recently to visualize spontaneous pulsations of central retinal vein in daily ophthalmic practice and also as a non-invasive means to assess intracranial pressure [24,25].

## 5. Imaging Techniques

Computed tomography (CT scans) and magnetic resonance imaging (MRI) are the preferred imaging modalities for the neuro-ophthalmic assessment [26,27]. The details are as follows.

### 5.1. Computed Axial Tomography Scan

CT scans, first introduced in 1972, is based on standard X-ray attenuation by tissues of various densities [28]. The CT X-ray source rotates around the patient, and the X-ray detectors located on the opposite side of the X-ray source measure the amount of attenuation. The computer analyzes the data points, and attenuation values are expressed in Hounsfield units assigned to each pixel, which are then compared to the attenuation value of water (zero) and reconstructed into a series of pixels which form images displayed on a computer screen. Dense materials like bone appear bright white, while less dense material like air appear darker. The rest are displayed in a gray-scale gradient. Iodinated contrast material can be used to improve the sensitivity and specificity of the CT scan interpretation [29]. However, in many situations they are contraindicated or not preferred (e.g., allergy, pregnancy).

Temporary blindness is a rare complication of preeclampsia and eclampsia. Cortical blindness has been reported in cases of pregnancy induced hypertension(PIH) [30]. This has been attributed to cerebral edema of occipital lobes that develops in severe cases of toxemia in all likelihood due to cerebral vascular spasm. The cerebral edema of the occipital lobe has been documented in such cases by CT scans. Complete visual recovery is common in such cases and usually parallels the resolution of cerebral edema. Not all cases have this favorable outcome, and some cases develop residual cerebral damage, as documented by electroencephalography [31]. Hysterical intracranial venous thrombosis and other causes of cortical blindness must be considered in pregnant patients with visual loss and normal fundii. The major ophthalmic indication for performing a venogram is to exclude dural venous sinus thrombosis in patients presenting with papilloedema from increased intracranial pressure.

Drayer and Rosenbaum confirmed that CT may demonstrate *in vivo* vasogenic, ischemic, cytotoxic or periventricular (interstitial) edema [32]. They state that CT findings of vasogenic cerebral edema consisted of regions of abnormally diminished attenuation co-efficients involving only white matter of brain. The most common CT findings are transient subcortical white matter hypodensities in the parieto-occipital areas of the brain. The lesions correspond to the watershed areas where the anterior, middle and posterior cerebral arteries meet [33]. An early breakthrough of cerebral auto-regulation occurs in watershed areas. Vasospasm is thought to cause local ischemia, arteriolar necrosis and disruption of the blood–brain barrier, which leads to cerebral edema. With disruption of the blood–brain barrier, vasogenic edema and pericapillary ring hemorrhages occur and has been proposed as one of the etiologies of seizures.

Single photon emission tomography (SPECT) scans showed perfusion deficits in watershed areas, the majority of which are in the parieto-occipital areas of the brain [34]. SPECT scans are superior in detecting neuropathophysiological alterations in eclampsia. The transient vasodialatory effect has been proposed to account for the increased cerebral blood flow velocities. It indicates the anatomical sites affected. SPECT scanning involves intravenous injection of radioisotopes and assesses alterations in the regional cerebral blood flow.

Positron emission tomography (PET) and SPECT both use radiolabelled molecules to image local metabolic changes. PET plays a limited role in imaging primary orbital disease [35]. PET is utilized in the assessment of patients with cortical blindness. Dynamic perfusion CT imaging is particularly useful in acute stroke [36]. A superior contrast resolution makes MRI more sensitive than CT scanning in the detection of cerebral abnormalities in eclampsia [37].

### 5.2. Magnetic Resonance Imaging (MRI)

MRI is indicated in unexplained visual loss, amurosis fugax, unilateral or bilateral optic neuropathy, cortical blindness, papillary defects, lid abnormalities, papilloedema, optic atrophy and headache [38]. MRI is given priority for almost all other neuro-ophthalmic indications because of superior soft tissue resolution of intracranial anatomy (e.g., meninges, cavernous sinus, posterior fosssa and dural venous sinus). MRI utilizes signal from the resonance within a large magnetic field. The static magnetic field is created by the MRI scanner in the unit Tesla (T). The detailed physics of MRI is beyond the scope of this review. MRI is based on signal intensity (*i.e.*, hyperintense, isointense or hypointense). In MRI, the two most common pulse sequences are T1- and T2-weighted images. (T1W1 and T2W2). On a T2-weighted scan, water and fluid-containing tissues are bright, and fat-containing tissues are dark; on T1-weighted images, the fluid is dark, and the fat is bright. T1-weighted images show excellent gray or white matter differentiation and spatial resolution providing detailed anatomic images. T2-weighted images provide more physiologic information and better detect pathological conditions.

#### 5.2.1. Imaging Sequences in MRI

Imaging sequences are ordered depending upon clinical indication, *i.e.*, fat suppression for orbital post contrast study [39], fluid attenuation inversion recovery (FLAIR) for white matter lesions [40], gradient recall echo (GRE) for hemorrhages [41], diffusion-weighted imaging for strokes [42,43] or posterior reversible encephalopathy syndrome (PRES) [44]. Posterior reversible encephalopathy syndrome (PRES) refers to pathologic conditions with headache, visual disorders, seizure, altered consciousness-conditions that regress both clinically and radiologically in a few weeks with the elimination of etiologic factors. PRES commonly occurs in preeclampsia/eclampsia.

In FLAIR, the free water signal can be attenuated and made dark, while water in edematous and pathologic tissues will remain bright [40]. Diffusion-weighted imaging (DWI) measures aberrancies in expected Brownian motion of free water [42]. The mobility of water within intracranial tissue is called the apparent diffusion coefficient (ADC). DWI/ADC is indicated in acute ischemic stroke where increased restriction of water diffusion can differentiate acute ischemic cytotoxic edema from vasogenic edema. Diffusion-weighted imaging is an MRI technique that is based on microscopic random Brownian motion of water. The changes in water molecular diffusion can be measured as signal intensity *in vivo* with DWI. DWI differentiates various stages of cerebral infarction. GRE MR sequences are useful for demonstrating hemorrhages associated with arteriovenous or cavernous malformations, intra or extra axial intracranial hemorrhages [41]. GRE depicts blood products more precisely. Fat suppression sequences allow a better visualization of the underlying pathological lesions on TIWI particularly when contrast is used. Perfusion weighted imaging (PWI) provides information regarding cerebro vascular hemodynamic parameters such as cerebral blood volume, time to peak, mean transit time and cerebral blood flow.

#### 5.2.2. Magnetic Resonance Spectroscopy

Magnetic resonance spectroscopy (MRS) is based on the detection of various proton MR spectra [45]. The commonly measured metabolites in MRS include N-acetylaspartate, (NAA), creatinine and phosphocreatinine (Cr), choline-containing phospholipids (Cho) and lactate. Other metabolites include glutamate, glutamine, gamma aminobutyric acid, myoinositol and fatty acids. MRS is indicated in ischemic and inflammatory conditions.

*In vivo* proton MR spectroscopy may help to differentiate cerebral edema from ischemia in patients with eclampsia and thus may help to determine the prognosis of these patients. In patients with acute cerebral ischemia, *in vivo* proton MR spectroscopy shows an increase in lactate less than 24 h after the onset of stroke, even when no conventional MR imaging abnormalities are detectable.

#### 5.2.3. FMRI, PET, and SPECT

Functional MRI (fMRI) studies might be particularly useful when structural imaging appears normal despite clinical findings that suggest underlying brain dysfunction [46]. The signal change of blood oxygenation level-dependent mechanisms can be imagined on fMRI.

PET and SPECT both use radiolabelled molecules to image local metabolic changes. Cranial MR imaging in women with eclampsia reveals characteristic multifocal curvilinear abnormalities at the gray-white matter junctions. MR imaging findings have also been confirmed by pathological examination. They are the hallmark of preeclampsia-eclamptic hypertensive encephalopathy. The lesions are reversible. These findings are collectively termed “posterior leukoencephalopathy syndrome.”

#### 5.2.4. Angiography

Contrast enhanced angiography and magnetic resonance angiography (MRA) have demonstrated reversible vasospasm of medium and large intracranial vessels [47,48]. Angiography is an invasive technique involving risks and complications. Conventional angiographic findings in patients of PIH include a reversible cerebral segmental arterial narrowing of large and medium vessels. These are due to intimal hyperplasia and reversible vasospasm. Recent studies demonstrate vasospasm of arteries of the Circle of Willis and extending peripherally. MR scanning with intravenous contrast material improves detection of pathology by demonstrating areas of blood–brain barrier breakdown. Gadolinium is preferred, as it is a non-iodinated, paramagnetic metal ion, as opposed to an iodinated contrast material utilized in CT scan.

The preferred technique for evaluating cerebral venous sinus thrombosis is pre- and post-contrast MRI with a contrast-enhanced magnetic resonance venography (MRV). There are two types of MRA: (1) time of flight (TOF) MRA and (2) phase-contrast (PC) MRA. Both can acquire data using a 2-D or 3-D scan. MRA is indicated in patients with ischemic attacks, transient visual loss, amurosis fugax and in patients with cortical strokes.

Newer MRI sequences such as CISS (constructive interference in steady state) and FIESTA (fast imaging employing steady state acquisition) have been deployed to better demonstrate the cranial nerves and might be particularly useful for smaller intrinsic lesions of the nerve and vascular abnormalities [49].

MR is superior to CT for most intracranial neuro-ophthalmic indications. Recent developments in vascular imaging, *i.e.*, MRA and CTA, have reduced the need for catheter angiography at many institutions.

The occipital lobe is the most affected region in PIH; parietal, frontal, temporal lobe and basal ganglion involvement follow this. The cerebellum and brain stem might be involved in more severe cases. Visual disorders, altered consciousness and convulsions were frequent in cases where cerebral edema was detected in MR imaging. Brain lesions were correlated with seizures. The detection of visual disorders, altered consciousness or convulsions in follow-up of PIH patients should be a warning for a possible brain lesion and an indication for MR imaging. Increased permeability of the blood–brain barrier related to endothelial injury is believed to play a major role in pathogenesis of preeclampsia/eclampsia.

Visual field loss patterns in pregnant women include bitemporal loss, concentric constriction and an enlarged blind spot and are attributed to serous retinal detachment, retinal hemorrhage, optic nerve head edema, nerve fiber layer infarcts and an involvement of occipital cortex and lesions of the central nervous system. The lesions were reversible. Visual evoked potentials, ERGs and electroencephalograms (EEGs) have also been used in conjunction to imaging techniques to understand the phenomena that precede eclampsia and also to ascertain the clinical findings [50,51,52,53].

To summarize the discussion, ophthalmologists should be aware of various modalities like MRA, CTA, MRV, CTV and specific sequences such as fat suppression, fluid attenuation inversion recovery (FLAIR), gradient recall echo imaging (GRE), diffusion-weighted imaging (DWI), perfusion-weighted imaging (PWI) and dynamic perfusion CT (PCT). In addition to functional imaging, *i.e.*, functional MRI (fMRI), positron emission tomography (PET) and single photon emission tomography (SPECT) are also available on hand for precision in diagnosis.

## 6. OCT

Optical coherence tomography (OCT) is non-contact, topographic, biomicroscopic device that provides high resolution, cross-sectional digital images of biological tissues *in vivo* and in real time [54]. OCT is similar to B-mode of ultrasound imaging, but it utilizes light instead of sound. It is based on the principles of low coherence intererometry. OCT is invaluable in diagnosis and in the follow-up of diseases involving anterior as well as posterior segments of the orbit, primary or secondary to systemic diseases as demonstrated in Figure 1, a case of serous retinal detachment in preeclamptic patient [7]. OCT is more than helpful when diagnosis is hampered by mild ocular media opacities like cataract, posterior capsular opacification, vitreous hemorrhage, asteroid hyalosis, vitritis, *etc.* Time domain OCT acquires A-scan by varying the length of the reference arm in an interferometer in such a way that the scanned length of the reference arm corresponds to the A-Scan length. Spectral domain OCT (SD-OCT), also known as the Fourier domain OCT, is a new high-definition, 3-D retinal micro structural imaging technology in which no mechanical scanning of the reference arm is needed. Spectral domain OCT can obtain fine retinal images with resolutions up to 5 μm. Spectral domain optical doppler tomography can measure retinal blood flow in pregnancy and preeclamptic states. Spectral domain OCT provides better identification of normal and pathological structures in patients with poor media clarity as compared to time domain OCT [55].

### Multinodal Imaging

Multinodal imaging offers the benefits of retinal angiography along with OCT that incorporates a fusion of confocal scanning laser ophthalmoscope and spectral domain OCT. Multinodal imaging facilitates infrared reluctance imaging, red free imaging, fluorescein angiography, ICGA, autofluorescence and OCT imaging. It offers the advantage of utilizing the individual imaging modalities or the imaging modalities simultaneously or in different combinations. Macular thickness parameters measured using OCT correlated with the degree of proteinuria in preeclampsia. These changes reversed soon after delivery. Abnormal OCT manifestations were correlated with contrast sensitivity tests in pregnancy-induced hypertensive states [56].

## 7. Ultrasonography

**Figure 3:**
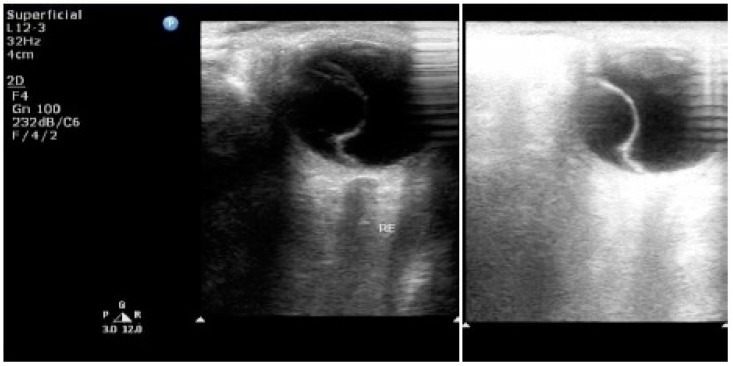
2-D ultrasonography of the right eye showing a detachment of the retina—serous variety [8]. Published with permissions of the publisher-Scholar Science Journals and International Journal of Biomedical Research. Original source: Tayade, S.; Wattamwar, A. Bilateral retinal detachment in pregnancy complicated by preeclampsia, eclampsia and placental abruption. *Int J Biomed Res* **2014**, *5*, 780–782, http://dx.doi.org/10.7439/ijbr.v5i12.818. This work is licensed under a Creative Commons Attribution 4.0 International License. http://creativecommons.org/licenses/by/4.0/.

Ultrasound has enabled examination of the eye and orbit in high-resolution with both A-Scan (time-amplitude) and B-scan (scanned, intensity modulated) techniques, doppler techniques and recently also 3-D approaches [57,58]. A-scan provides a rapid examination of the globe by utilizing quantitation and kinetic studies. B-scan ultrasonographic imaging gives an anatomical display similar to a section of the eye and orbit. Ultrasonographic can examine eyes which cannot be visualized because of corneal opacities, cataract, hemorrhage, *etc.* Newer modifications include contrast-enhanced ultrasound (CEUS) and 3-D reformatting of ultrasound images for accuracy of diagnosis in ophthalmology. It is more than helpful in differentiating retinal detachment from vitreous membrane. Figure 3 demonstrates the utility of 2-D ultrasonography for diagnosis in a patient with hypertensive disease of pregnancy with serous retinal detachment [8]. It is cost-effective and can be easily performed when CT scans and MRI are contraindicated. Unique for ophthalmology is the newly invented, highly resolving so-called “ultrasound biomicroscopy,” utilizing ultrasound frequencies of 50 MHz and higher. Ultrasound is valuable, however, in particular as part of the initial clinical work-up, and for the follow-up of orbital disease. Color doppler imaging, tissue characterization, parameter image staining and 3-D volume rendering are new and useful adjuncts to ocular diagnosis.

## 8. Doppler Velocimetry of the Ophthalmic Artery

Doppler examination of the retinal vasculature is beneficial in evaluation, treatment and management of hypertension during pregnancy [59,60]. It is easy to perform, non-invasive and inexpensive. Using the velocity wave, maximum systolic and minimum diastolic blood flow velocities were assessed in cm/second. The resistance index (RI) was calculated (systolic max minus diastolic minimum divided by maximum systolic velocity). The peak ratio (PR), a ratio between the peak diastolic velocity (after the protodiastolic notch) and the initial peak (peak systolic velocity) is an index of dicrotic waves which allows analysis of flow velocity wave elevation during the mesodiastole and provides good quantification of specific changes in dicrotic waves. The higher the peak diastolic velocity, the higher the PR value. The pulsability index (PI) and velocity are necessary to evaluate blood circulatory change. The pulsability index in preeclamptic women was lower than in women with no visual symptoms. A decreasing PI value in preeclamptic women suggests a decreased vascular resistance or hyperperfusion of orbital vessels. A decrease in orbital vascular resistance and increase in orbital perfusion are stronger in patients with visual symptoms and retinal edema. Preeclamptic women have orbital vascular vasodilatation or hyperperfusion. In patients with mild, moderate preeclampsia, ophthalmic artery doppler ultrasonography detects hemodynamic changes. Increased flow velocity measurements in the middle and posterior cerebral arteries were found in the majority. Thus, transcranial doppler (TCD) ultrasonography is a non-invasive technique to assess changes in the velocity of cerebral blood flow in accordance with changes in blood vessel diameter and caliber.

## 9. Laboratory Investigations

Patients with hypertensive disorders had abnormalities of lactic dehydrogenase, alkaline phosphatase, serum glutamic oxaloacetic transaminase (SGOT), uric acid, blood urea nitrogen (BUN) and creatinine. The recommended laboratory tests are a complete blood count (blood smear and platelet count), a coagulation profile and serum creatinine tests. Liver function tests, cardiac function tests and other investigations are recommended if there is clinical evidence of an additional disease process [61,62,63,64]. The following tests, *i.e.* prothrombin time, activated partial thromboplastin time, platelet count, serum fibrinogen, fibrin degradation products and plasma fibrinogen used to detect coagulation disorders. Tests to assess renal functions include uric acid, creatinine, albumin, and blood urea nitrogen (BUN). Total bilirubin, alkaline phosphatase, lactic dehydrogenase and serum glutamic oxaloacetic transaminase (SGOT) are used to evaluate hepatic function.

Proteinuria is the most common investigation performed by health care professionals in primary and secondary health care centers to monitor pregnancy and predict its complications [63]. Urine analysis by visual reagent strip tests, total protein estimation in 24 h urine sample, spot urine protein: creatinine ratio and urine albumin to the creatinine ratio are the most frequently used laboratory protocols in antenatal clinics and community centers. Several studies elaborate that proteinuria has been associated with an increase in maternal and fetal mortality and morbidity. Microalbuminuria refers to a sub-clinical elevation of urinary albumin excretion [65]. In predicting hypertensive disorder of pregnancy, urinary microalbumin excretion may be a better option compared to conventional tests.

Examinations of CO_2_ combining power, NPN and uric acid blood levels as well as the amount of albumin in urine was made before and after delivery. If a consistent relationship between the above factors and retinal arteriolar findings could be found, it would prove most helpful in determining when interruption of pregnancy should be done. There were no significant findings in the Grade O or Grade I retinal arteriolar changes in mild preeclampsia. However, in Grade II classification, when dead infants were delivered, the uric acid and NPN increased above normal, while CO_2_ combining power dropped below normal one or two weeks before delivery. After delivery, the blood values returned to normal levels in one to three weeks. Thus, definite blood chemistry changes, along with retinal arteriolar changes, are usually consistent enough to be reliable as an index to indicate proper time for interruption. Our recent experience indicates that such a practice has definitely increased the number of living babies.

Studies have been carried out to correlate serum uric acid levels [66], circulating NO metabolites [67], baseline serum and cerebrospinal fluid magnesium levels [68], serum levels of leptins, cytokines, and lipoproteins [69], B-type natriuretic peptide (BNP) [70], *etc.*, with maternal and fetal morbidity. Pre-eclampsia is associated with an increase in serum levels of leptin, TNF-A, cytokines, triglycerides, total cholesterol and LDL-cholesterol along with a significant reduction in serum levels of HDL-cholesterol and Apo-A. The association was attributed to an abnormal lipid metabolism, immune activation involved in the pathogenesis of the disease. Recently, fibronectin has emerged as a promising marker for prediction of hypertensive disorders in pregnancy [71]. Attempts have been made to correlate the tests with maternal or fetal outcomes.

## 10. Ophthalmoscopy

Ophthalmoscopy is the traditional and most economical method to assess the retinal changes and to guide the obstetrician regarding the termination of pregnancy and induction of labor [4,72]. Ophthalmoscopy is also of extreme utility in medical centers with compromised resources and in third-world countries.

Ophthalmoscopy can easily detect retinal arteriolar changes, hemorrhages, exudates, retinal detachment, disc changes, *etc.* Termination of pregnancy is indicated if angiospasm persists in spite of medical management. Hemorrhagic retinitis, retinal detachment and papilloedema are an indication for termination of pregnancy. In complicated cases with extensive hemorrhagic retinitis, cotton wool spots and/or exudates, special monitoring by the obstetrician is required. The investigations discussed in this review, *i.e.*, angiography, imaging, doppler ultrasound, fluorophotometry, ophthalmodynamometry, biochemistry, *etc.*, are for a precision in diagnosis, in management and in advanced research in the etiopathology of the disease process and improvement in the maternal and child health care delivery systems. Neuro-ophthalmic intervention without proper imaging is not possible. Ophthalmodynamometry and fluorophotometry have opened up new dimensions in nano-medicine and the drug delivery and assessment of intracranial pressure, with more precision than the detection of papilloedema at later stages via ophthalmoscopy. Laboratory analysis with new biomarkers might detect the disease process prior to fundus changes.

## 11. Conclusions

Specialized investigation modalities have helped us to understand the pathogenesis of hypertensive disorders of pregnancy. The harmonious integration of investigative modalities with current developments in holograms and telemedicine would provide a helping hand in clinical diagnosis, the prevention of complications, the monitoring of treatment regimes and follow-up. They have been successful in reducing maternal and fetal mortality and morbidity attributed to hypertensive disorders of pregnancy [73,74].

## References

[1] Davey D.A., MacGillivray I. (1988). The classification and definition of the hypertensive disorders of pregnancy. Am. J. Obstet. Gynecol..

[2] Duley L. (1992). Maternal mortality associated with hypertensive disorders of pregnancy in Africa, Asia, Latin America and the Caribbean. Br. J. Obstet. Gynaecol..

[3] Brown M.A., Buddle M.L. (1996). Hypertension in pregnancy: Maternal and fetal outcomes according to laboratory and clinical features. Med. J. Aust..

[4] Seidman D.S., Serr D.M., Ben-Rafael Z. (1991). Renal and ocular manifestations of hypertensive diseases of pregnancy. Obstet. Gynecol. Surv..

[5] Conde-Agudelo A., Lede R., Belizán J. (1994). Evaluation of methods used in the prediction of hypertensive disorders of pregnancy. Obstet. Gynecol. Surv..

[6] Schreyer P., Tzadok J., Sherman D.J., Herman A., Bar-Itzhak R., Caspi E. (1991). Fluorescein angiography in hypertensive pregnancies. Int. J. Gynaecol. Obstet..

[7] Sreckovic S.B., Janicijevic-Petrovic M.A., Stefanovic I.B., Petrovic N.T., Šarenac T.S., Paunovic S.S. (2011). Bilateral retinal detachment in a case of preeclampsia. Bosn. J. Basic Med. Sci..

[8] Tayade S., Wattamwar A. (2014). Bilateral retinal detachment in pregnancy complicated by preeclampsia, eclampsia and placental abruption. Int. J. Biomed. Res..

[9] Fastenbery D.M., Fetkenhour C.L., Choromokos E., Shoch D.E. (1980). Choroidal vascular changes in toxaemia of pregnancy. Am. J. Ophthalmol..

[10] Sood P., Saxena N., Talwar D. (2015). OCT angiography: An upcoming tool for diagnosis and treatment of retinal vascular diseases. DJO.

[11] Valluri S., Adelberg D.A., Curtis R.S., Olk R.J. (1996). Diagnostic indocyanine green angiography in preeclampsia. Am. J. Ophthalmol..

[12] Wolf S., Wald K.J., Elsner A.E., Staurenghi G. (1993). Indocyanine green choroidal video angiography: A comparison of imaging analysis with the scanning laser ophthalmoscope and the fundus camera. Retina.

[13] Hope-Ross M., Yannuzzi L.A., Gragoudas E.S., Guyer D.R., Slakter J.S., Sorenson J.A., Krupsky S., Orlock D.A., Puliafito C.A. (1994). Adverse reactions due to indocyanine green. Ophthalmology.

[14] Fineman M.S., Maguire J.I., Fineman S.W., Benson W.E. (2001). Safety of indocyanine green angiography during pregnancy: A survey of the retina, macula, and vitreous societies. Arch. Ophthalmol..

[15] Freeman W.R. (1998). Simultaneous indocyanine green and fluorescein angiography using a confocal scanning laser ophthalmoscope. Arch. Ophthalmol..

[16] Kayazawa F., Miyake K. (1984). Ocular fluorophotometry in patients with essential hypertension. Arch. Ophthalmol..

[17] Chaine G., Attali P., Gaudric A., Colin M.C., Quentel G., Coscas G. (1986). Ocular fluorophotometric and angiographic findings in toxemia of pregnancy. Arch. Ophthalmol..

[18] Raines M.F. (1986). Vitreous fluorophotometry. Semin. Ophthalmol..

[19] Tyagi P., Kadam R.S., Kompella U.B. (2012). Comparison of suprachoroidal drug delivery with subconjuctival and intravitreal routes using noninvasive fluorophotometry. PLoS ONE.

[20] Wunsh S.E. (1969). Ophthalmodynamometry. N. Eng. J. Med..

[21] Koch F.L. (1941). Retina in systemic vascular hypertension: A clinical study of the calibre of the retinal arterioles and the retinal arterial diastolic blood pressure. Arch. Ophthalmol..

[22] Van dar Worllff T.J. (1972). The pressure measured in ophthalmodynamometry. Arch. Ophthalmol..

[23] Stodtmeister R., Oppitz T., Spoerl E., Haustein M., Boehm A.G. (2010). Contact lens dynamometry: The influence of age. Investig. Ophthalmol. Vis. Sci..

[24] Legler U., Jonas J.B. (2007). Assessment of the spontaneous pulsations of the central retinal vein in daily ophthalmic practice. Clin. Exp. Ophthalmol..

[25] Firsching R., Müller C., Pauli S.U., Voellger B., Röhl F.W., Behrens-Baumann W. (2011). Noninvasive assessment of intracranial pressure with venous ophthalmodynamometry: Clinical article. J. Neurosurg..

[26] Lee A.G., Johnson M.C., Policeni B.A., Smoker W.R. (2009). Imaging for neuro-ophthalmic and orbital disease—A review. Clin. Exp. Ophthalmol..

[27] Duncan R.O., Hadley D., Bone I., Symonds E.M., Worthington B.S., Rubin P.C. (1989). Blindness in eclampsia: CT and MR imaging. J. Neurol. Neurosurg. Psychiatry.

[28] Vellody D., Waldron R.L., Abbott D.C. (1985). Computed tomography in preeclampsia-eclampsia syndrome. AJNR Am. J. Neuroradiol..

[29] Lee A.G., Hayman L.A., Ross A.W. (2000). Neuroimaging contrast agents in ophthalmology. Surv. Ophthalmol..

[30] Lan S.P.C., Chan F.L., Yu Y.L., Woo E., Huang C.Y. (1987). Cortical blindness in toxaemia of pregnancy: Findings on computed tomography. Br. J. Radiol..

[31] Moodley J., Bobat S.M., Hoffman M., Bill P.L.A. (1993). Electroencephalogram and computerized cerebral tomography findings in eclampsia. Br. J. Obstet. Gynaecol..

[32] Drayer B.P., Rosenbaum A.E. (1979). Brain edema defined by cranial computed tomography. J. Comput. Assist. Tomogr..

[33] Zunker P., Ley-Pozo J., Louwen F., Schuierer G., Holzgreve W., Ringelstein E.B. (1995). Cerebral hemodynamics in preeclampsia/eclampsia syndrome. Ultrasound Obstet. Gynecol..

[34] Schwartz R.B., Jones K.M., Kalina P., Bajakian R.L., Mantello M.T., Garada B., Holman B.L. (1992). Hypertensive encephalopathy: Findings on CT, MR imaging, and SPECT imaging in 14 cases. AJR Am. J. Roentgenol..

[35] Tai Y.F., Pioccini P. (2004). Applications of positron emission tomography (PET) in neurology. J. Neurol. Neurosurg. Psychiatry.

[36] Mayer T.E., Hamann G.F., Baranczyk J., Rosengarten B., Klotz E., Wiesmann M., Missler U., Schulte-Altedorneburg G., Brieckmann H.J. (2000). Dynamic CT perfusion imaging of acute stroke. AJNR Am. J. Neuroradiol..

[37] Dahmus M.A., Barton J.R., Sibai B.M. (1992). Cerebral imaging in eclampsia: Magnetic resonance imaging *versus* computed tomography. Am. J. Obstet. Gynecol..

[38] Digre K.B., Varner M.W., Osborn A.G., Crawford S. (1993). Cranial magnetic resonance imaging in severe preeclampsia *vs.* eclampsia. Arch. Neurol..

[39] Delfaut E.M., Beltran J., Johnson G., Rousseau J., Marchandise X., Cotten A. (1999). Fat suppression in MR imaging: Techniques and pitfalls. Radiographics.

[40] Arakia Y., Ashikaga R., Fujii K., Nishimura Y., Ueda J., Fujita N. (1999). MR fluid-attenuated inversion recovery imaging as routine brain T2-weighted imaging. Eur. J. Radiol..

[41] Wycliffe N.D., Choe J., Holshouser B., Oyoyo U.E., Haacke E.M., Kido D.K. (2004). Reliability in detection of hemorrhage in acute stroke by a new 3-D gradient recalled echo susceptibility-weighted imaging technique compared to computed tomography: A retrospective study. J. Magn. Reson. Imaging.

[42] Valentini V., Gaudino S., Spagnolo P. (2003). Diffusion and perfusion MR imaging. Rays.

[43] Gregory D.G., Pelak V.S., Bennett J.L. (2003). Diffusion-weighted magnetic resonance imaging and the evaluation of cortical blindness in preeclampsia. Surv. Ophthalmol..

[44] Lamy C., Oppenheim C., Meder J.F., Mas J.L. (2004). Neuroimaging in posterior reversible encephalopathy syndrome. J. Neuroimaging.

[45] Castillo M., Kwock L., Mukherji S.K. (1996). Clinical applications of proton MR spectroscopy. AJNR Am. J. Neuroradiol..

[46] Gore J.C. (2003). Principles and practice of functional MRI of the human brain. J. Clin. Investig..

[47] Ito T., Sakai T., Inagawa S., Utsu M., Bun T. (1995). MR angiography of cerebral vasospasm in preeclampsia. AJNR Am. J. Neuroradiol..

[48] Goldman J.P. (2003). New techniques and applications for magnetic resonance angiography. Mt. Sinai J. Med. N. Y..

[49] Kulkami M. (2011). Constructive interference in steady-state/FIESTA-C clinical applications in neuroimaging. J. Med. Imaging Radiat. Oncol..

[50] Marsh M.S., Smith S. (1994). The visual evoked potential in the assessment of central nervous system effects of preeclampsia: A pilot study. Br. J. Obstet. Gynaecol..

[51] Thomas S.V., Somanathan N., Radhakumari R. (1995). Interictal EEG changes in eclampsia. Electroencephalogr. Clin. Neurophysiol..

[52] Kwok A.H., Li J., Lai T.Y., Chan W.M., Bhede P., Lam D.S. (2001). Multifocal electroretinographic and angiographic changes in preeclampsia. Br. J. Ophthalmol..

[53] Citirik M., Tulay S., Zilelioglu O. (2008). Bilateral permanent concentric visual field defect secondary to severe preeclampsia. Clin. Ophthalmol..

[54] Theodossiadis P.G., Kollia A.K., Gogas P., Panagiotidis D., Moschos M., Theodossiadis G.P. (2002). Retinal disorders in preeclampsia studied with optical coherence tomography. Am. J. Ophthalmol..

[55] Leitgeb R., Hitzenberger C.K., Fercher A.F. (2003). Performance of fourier domain *vs.* time domain optical coherence tomography. Opt. Expess.

[56] Wang Z., Zou Y., Li W., Wang X., Zhang M., Wang W. (2015). Application of optical coherence tomography and contrast sensitivity test for observing fundus changes of patients with pregnancy-induced hypertension syndrome. Medicine.

[57] Fledelius H.C. (1997). Ultrasound in ophthalmology. Ultrasound Med. Biol..

[58] Sconfienza L.M., Lacelli F., Ardemagni A., Perrone N., Bertolotto M., Padolecchia R., Serafini G. (2010). High-resolution, 3-D, and contrast-enhanced ultrasonographic findings in diseases of the eye. J. Ultrasound.

[59] Williams K.P., MacLean C. (1993). Maternal cerebral vasospasm in eclampsia assessed by transcranial Doppler. Am. J. Perinatol..

[60] Riskin-Mashiah S., Belfort M.A., Saade G.R., Herd J.A. (2002). Transcranial doppler measurement of cerebral velocity indices as a predictor of preeclampsia. Am. J. Obstet. Gynecol..

[61] Waugh J., Bell S.C., Kilby M.D., Lambert P., Shennan A., Halligan A. (2005). Urine protein estimation in hypertensive pregnancy: Which thresholds and laboratory assay best predict clinical outcome?. Hypertens. Pregnancy.

[62] Martin J.N., May W.L., Magann E.F., Terrone D.A., Rinehart B.K., Blake P.G. (1999). Early risk assessment of severe preeclampsia: Admission battery of symptoms and laboratory tests to predict likelihood of subsequent significant maternal morbidity. Am. J. Obstet. Gynecol..

[63] Newman M.G., Robichaux A.G., Stedman C.M., Jaekle R.K., Fontenot M.T., Dotson T., Lewis T.F. (2003). Perinatal outcomes in preeclampsia that is complicated by massive proteinuria. Am. J. Obstet. Gynecol..

[64] Melchiorre K., Thilaganathan B. (2011). Maternal cardiac function in preeclampsia. Curr. Opin. Obstet. Gynecol..

[65] Salako B.L., Olayemi O., Odukogbe A.T., Adedapo K.S., Aimakhu C.O., Alu F.E., Ola B. (2004). Microalbuminuria in pregnancy as a predictor of preeclampsia and eclampsia. West Afr. J. Med..

[66] Voto L.S., Illia R., Darbon-Grosso H.A., Imaz F.U., Margulies M. (1988). Uric acid levels: A useful index of the severity of preeclampsia and perinatal prognosis. J. Perinat. Med..

[67] Nishizawa H., Pryor-Koishi K., Suzuki M., Kato T., Sekiya T., Tada S., Kurahashi H., Udagawa Y. (2009). Analysis of nitric oxide metabolism as a placental or maternal factor underlying the etiology of pre-eclampsia. Gynecol. Obstet. Investig..

[68] Fong J., Gurewitsch E.D., Volpe L., Wagner W.E., Gomillion M.C., August P. (1995). Baseline serum and cerebrospinal fluid magnesium levels in normal pregnancy and preeclampsia. Obstet. Gynecol..

[69] Koçyıgıt Y., Atamer Y., Atamer A., Tuzcu A., Akkus Z. (2004). Changes in serum levels of leptin, cytokines and lipoprotein in preeclamptic and normotensive pregnant women. Gynecol. Endocrinol..

[70] Resnik J.L., Hong C., Resnik R., Kazanegra R., Beede J., Bhalla V., Maisel A. (2005). Evaluation of B-type natriuretic peptide (BNP) levels in normal and preeclamptic women. Am. J. Obstet. Gynecol..

[71] Leeflang M.M., Cnossen J.S., Van der Post J.A.M., Mol B.W., Khan K.S., Ter Riet G. (2007). Accuracy of fibronectin tests for the prediction of preeclampsia: A systematic review. Eur. J. Obstet. Gynecol. Reprod. Biol..

[72] Mussey R.D., Mundell B.J. (1939). Retinal examinations: A guide in the management of the toxic hypertensive syndrome of pregnancy. Am. J. Obstet. Gynecol..

[73] Perlmutter R., Friedland S. (1983). Computer-generated holograms in biology and medicine. IEEE Comput. Graph. Appl..

[74] Perednia D.A., Allen A. (1995). Telemedicine technology and clinical applications. JAMA.

